# Identification and Characterization of Bifunctional Proline Racemase/Hydroxyproline Epimerase from Archaea: Discrimination of Substrates and Molecular Evolution

**DOI:** 10.1371/journal.pone.0120349

**Published:** 2015-03-18

**Authors:** Seiya Watanabe, Yoshiaki Tanimoto, Hisashi Nishiwaki, Yasuo Watanabe

**Affiliations:** Faculty of Agriculture, Ehime University, Matsuyama, Ehime, 790–8566, Japan; University of Edinburgh, UNITED KINGDOM

## Abstract

Proline racemase (ProR) is a member of the pyridoxal 5’-phosphate-independent racemase family, and is involved in the Stickland reaction (fermentation) in certain clostridia as well as the mechanisms underlying the escape of parasites from host immunity in eukaryotic *Trypanosoma*. Hydroxyproline epimerase (HypE), which is in the same protein family as ProR, catalyzes the first step of the *trans*-4-hydroxy-L-proline metabolism of bacteria. Their substrate specificities were previously considered to be very strict, in spite of similarities in their structures and catalytic mechanisms, and no racemase/epimerase from the ProR superfamily has been found in archaea. We here characterized the ProR-like protein (OCC_00372) from the hyperthermophilic archaeon, *Thermococcus litoralis* (TlProR). This protein could reversibly catalyze not only the racemization of proline, but also the epimerization of 4-hydroxyproline and 3-hydroxyproline with similar kinetic constants. Among the four (putative) ligand binding sites, one amino acid substitution was detected between TlProR (tryptophan at the position of 241) and natural ProR (phenylalanine). The W241F mutant showed a significant preference for proline over hydroxyproline, suggesting that this (hydrophobic and bulky) tryptophan residue played an importance role in the recognition of hydroxyproline (more hydrophilic and bulky than proline), and substrate specificity for hydroxyproline was evolutionarily acquired separately between natural HypE and ProR.　A phylogenetic analysis indicated that such unique broad substrate specificity was derived from an ancestral enzyme of this superfamily.

## Introduction

**Fig 1:**
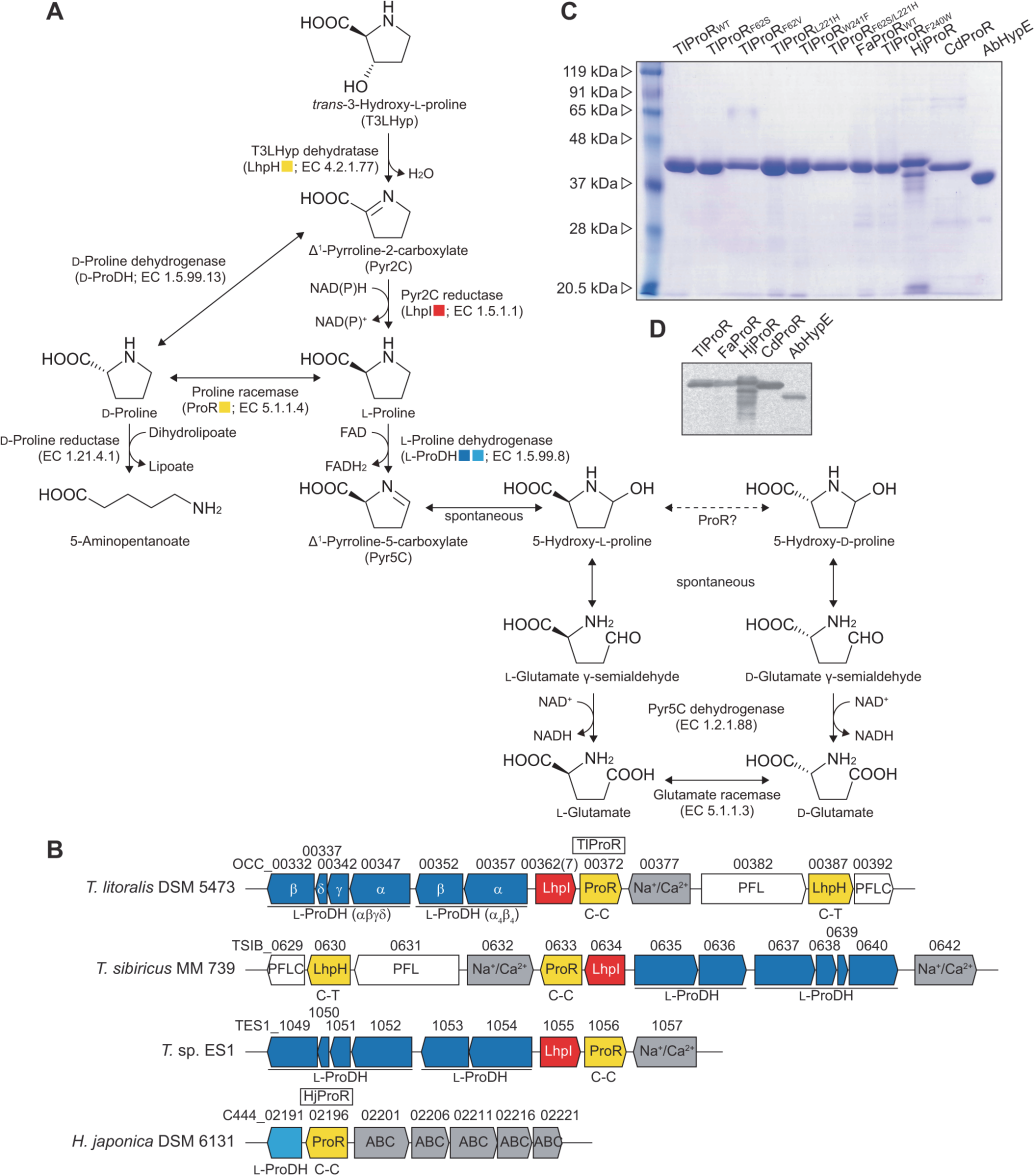
Putative metabolic network of proline and hydroxyproline in archaea. (A) Metabolic networks of L-proline, D-proline, and T3LHyp. (B) Schematic gene clusters related to the metabolism of proline and/or T3LHyp by archaea. Homologous genes are indicated in the same color and correspond to Fig. 1A. Putative genes in the box were purified and characterized in this study (see Fig. 1C). No clustering was found around the *FaProR* gene. C-C and C-T indicate a pair of catalytic amino acid residues of ProR superfamily enzymes. Gray putative genes are sequentially similar to other (amino acid) transporters. (C) Purification of recombinant His_6_ tag proteins. Five micrograms each of the purified protein were applied to a 12% (w/v) gel. (D) Western blot analysis. One microgram each of the purified protein were applied to a 12% (w/v) gel.

L-Proline can serve as a complete source of carbon and energy or of nitrogen for organisms. The metabolism of L-proline is generally initiated through its oxidation by L-proline dehydrogenase (EC 1.5.99.8; L-ProDH) to form Δ^1^-pyrroline-5-carboxylate (Pyr5C) [1] (Fig. 1A). Following its spontaneous hydrolysis, the L-glutamate γ-semialdehyde produced is then oxidized to L-glutamate by Pyr5C dehydrogenase (EC 1.2.1.88). L-Proline is alternatively metabolized to 5-aminopentanoate through two enzymatic steps. L-Proline racemase (EC 5.1.1.4; ProR) first catalyzes the racemization of L-proline to D-proline, followed by reductive cleavage to yield 5-aminopentanoate by D-proline reductase (EC 1.21.4.1). This pathway is only operative in certain clostridia including *Clostridium sticklandii* [2] and *Clostridium difficile* [3], clinically significant nosocomial pathogens, and *Trypanosoma* species including *Trypanosoma cruzi* [4, 5] and *Trypanosoma vivax* [6], the causative agents of Chagas disease and animal trypanosomiasis, respectively. In the former, the pathway is involved in the so-called “Stickland reaction (fermentation)”, whereas ProR in the latter has been implicated in the mechanisms underlying the escape of parasites from host immunity as a B-cell mitogen.

**Fig 2:**
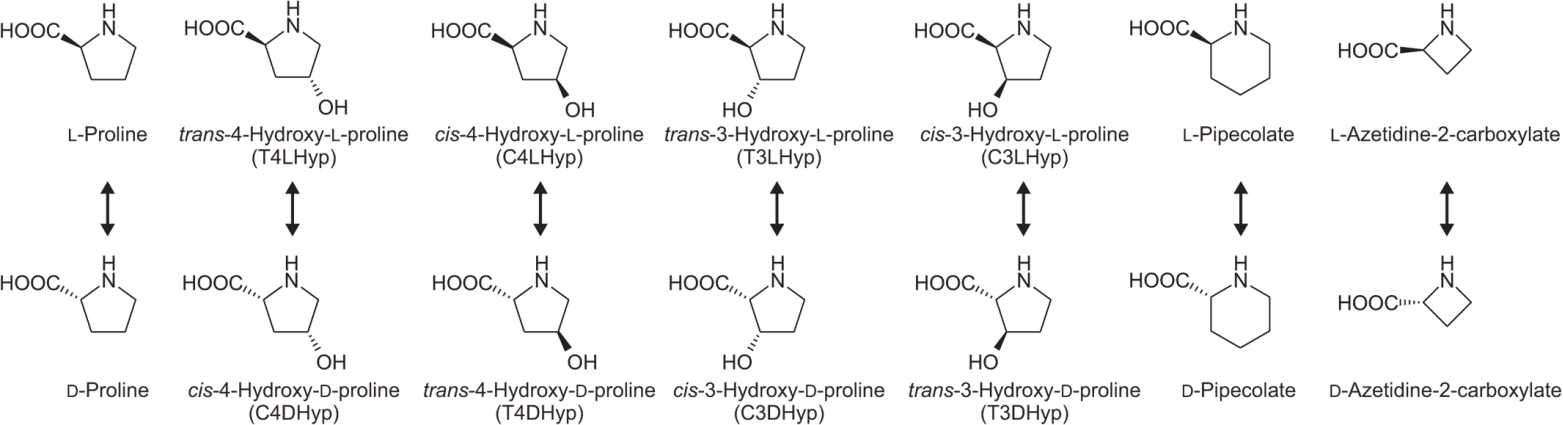
Library of proline derivatives. TlProR can enable the reversible racemization and epimerization of all proline derivatives (arrows).

Hydroxyproline has been detected in certain proteins, especially collagen, as well as some peptide antibiotics. In mammalian systems [1], the L-proline residue in procollagen is post-translationally hydroxylated to *trans*-4-hydroxy-L-proline (T4LHyp) or *trans*-3-hydroxy-L-proline (T3LHyp). Among the several stereoisomers of hydroxyproline including T4LHyp and T3LHyp (Fig. 2), T4LHyp is the most common in nature. In contrast to mammalians [1], some bacteria have been shown to metabolize T4LHyp to α-ketoglutarate via three intermediates through four enzymatic steps [7, 8]. Of these, hydroxyproline 2-epimerase (HypE; EC 5.1.1.8) first catalyzes the isomerization of T4LHyp to *cis*-4-hydroxy-D-proline (C4DHyp), which is then converted to Δ^1^-pyrroline-4-hydroxy-2-carboxylate (Pyr4H2C) by C4DHyp dehydrogenase (D-HypDH). In the metabolism of T3LHyp (Fig. 1A), T3LHyp dehydratase (EC 4.2.1.77; T3LHypD) initially catalyzes the dehydration of T3LHyp to Δ^1^-pyrroline-2-carboxylate (Pyr2C) via a putative Δ^2^-pyrroline-2-carboxylate intermediate [9]. The Pyr2C is then converted by NAD(P)H-dependent Pyr2C reductase (EC 1.5.1.1) to yield L-proline, which is metabolized through the general degradation of L-proline described above. This pathway is most commonly found in mammals [9], bacteria [10] and archaea [11].

Racemase enzymes catalyze the deprotonation/reprotonation of the chiral carbon (Cα) of both amino acid enantiomers, resulting in the stereoinversion of chiral centers. ProR is a member of the pyridoxal 5’-phosphate (PLP)-independent racemase family along with HypE and T3LHypD (although the reaction involving the latter is not epimerization) [9, 12, 13]. These enzymes have been classified into three types based on the two specific residues at the active sites [9]: the Cys-Cys type (ProR and HypE), Cys-Thr type (T3LHypD), and Ser-Cys or Ser-Thr type (function unknown). In the cases of ProR and HypE, catalysis is based on the same 1,1-proton transfer mechanism using two general acidic/basic cysteine residues located on opposite faces of the active site [12]. On the other hand, their substrate specificities were previously considered to be very strict: proline for ProR [13] and T4LHyp and *cis*-4-hydroxy-L-proline (C4LHyp) for HypE [13, 14]. Although the catalytic mechanism of T3LHypD currently remains unclear, dehydration may start by the abstraction of a proton from the Cα of the substrate by an active site cysteine residue as a general basic catalyst [9]. This enzyme can also only utilize T3LHyp as a substrate.

As described above, no racemase/epimerase from the ProR superfamily had been found in archaea. We here identified and characterized a ProR-like protein with a pair of Cys-Cys as (putative) active sites from the hyperthermophilic marine archaeon, *Thermococcus litoralis* [15], which differed from the previously isolated T3LHypD enzyme [11]. This protein could reversibly catalyze not only the racemization of proline, but also the epimerization of 4-hydroxyproline and 3-hydroxyproline with similar kinetic constants. Site-directed mutagenesis revealed several important amino acid residue(s) responsible for discriminating between proline and hydroxyproline. A phylogenetic analysis indicated that such unique broad substrate specificity was derived from an ancestral enzyme of this superfamily. To the best of our knowledge, this is the first study on the bifunctional enzyme of ProR and HypE from archaea.

## Materials and Methods

### Materials

T3LHyp was purchased from Kanto Chemical (Tokyo, Japan). C4LHyp, L-pipecolate, and D-pipecolate were obtained from Tokyo Chemical Industry (Tokyo, Japan). T4DHyp and C3LHyp were from Sigma Aldrich (USA). T4LHyp, C4DHyp, L-proline, and D-proline were from Wako Pure Chemical Industries (Osaka, Japan).

### General procedures

Basic recombinant DNA techniques were performed as described by Sambrook *et al*. [16]. Archaeal genomic DNA was prepared using a DNeasy Tissue Kit (Qiagen). PCR was carried out using a GeneAmp PCR System 2700 (Applied Biosystems) for 30 cycles in 50 μl of reaction mixture containing 1 U of KOD FX DNA polymerase (TOYOBO), appropriate primers (15 pmol), and template DNA under the following conditions: denaturation at 98°C for 10 s, annealing at 50°C for 30 s, and extension at 68°C for time periods calculated at an extension rate of 1 kbp∙min^−1^. DNA sequencing was carried out using the BigDye Cycle Sequencing Kit ver.3.1 (Applied Biosystems) and appropriate primers with the Genetic Analyzer 3130 (Applied Biosystems). High-pressure liquid chromatography (HPLC) was performed using an Agilent 1120 Compact LC system (TOSOH). Protein concentrations were determined by the method of Lowry et al. [17] with bovine serum albumin as the standard. SDS-PAGE was performed as previously described by Laemmli [18]. Western blot analysis was carried out using an ECL Western Blotting Analysis System (GE Healthcare) and RGS∙His HRP antibody (horseradish peroxidase-fused mouse monoclonal antibody against Arg-Gly-Ser-His_6_ in the N-terminal additional peptide of the expressed recombinant proteins (Qiagen))

### Plasmid construction for expression of recombinant proteins

The primer sequences used in this study are shown in S1 Table. In this study, the prefixes Tl (*T*. *litoralis*), Fa (*Ferroplasma acidiphilum*), Hj (*Haloarcula japonica*), Cd (*C*. *difficile*), and Ab (*Azospirillum brasilense*) were added to gene symbols or protein designations where necessary for clarity. The *TlProR* (GenBank accession number OCC_00372), *FaProR* (FACI_IFERC00001G0786), *HjProR* (C444_02196), *CdProR* (CD630_32370), and *AbHypE* genes (BAN78527) were amplified by PCR using primers containing appropriate restriction enzyme sites at the 5’- and 3’-ends and the genomic DNA of *T*. *litoralis* DSM 5473, *F*. *acidiphilum* DSM 12658, *H*. *japonica* DSM 6131, *C*. *difficile* 630, or *A*. *brasilense* ATCC 29145 as a template, respectively. Each amplified DNA fragment was introduced into BamHI-SalI sites (for the *TlProR*, *FaProR*, and *CdProR* genes), BamHI-PstI (for the *HjProR* gene), or BamHI-HindIII (for the *AbHypE* gene) in pETDuet-1 (Novagen), a plasmid vector used to confer an N-terminal His_6_ tag on expressed proteins, to obtain pET/TlProR_WT_, pET/FaProR_WT_, pET/HjProR, pET/CdProR, and pET/AbHypE_WT_, respectively.

In the present study, we used *F*. *acidiphilum* DSM 12658 instead of *Ferroplasma acidarmanus* fer1 as a target microorganism, for which there was 99% identity in the 16S rRNA sequence. We successfully amplified the *FaProR* gene by genomic PCR using oligonucleotide primers designed from the *F*. *acidarmanus* fer1 genome sequence. Unless otherwise noted, *Ferroplasma* sp. hereafter indicates strain DSM 12658.

### Expression and purification of His_6_-tagged recombinant proteins

*Escherichia coli* BL21-CodonPlus(DE3)-RIL (Novagen) harboring the constructed pETDuet-1 plasmid was grown at 37°C to a turbidity of 0.6 at 600 nm in Super broth medium (pH 7.0, 12 g tryptone, 24 g yeast extract, 5 ml glycerol, 3.81 g KH_2_PO_4_, and 12.5 g K_2_HPO_4_ per liter) containing 50 mg/liter ampicillin. After the addition of 1 mM isopropyl-β-D-thiogalactopyranoside (IPTG), the culture was grown for a further 6 h to induce the expression of the His_6_-tagged protein. The grown cells were harvested by centrifugation at 30,000 × *g* for 20 min, suspended in Buffer A (50 mM sodium phosphate buffer (pH 8.0) containing 300 mM NaCl and 10 mM imidazole), disrupted by sonication for 20 min at appropriate intervals on ice using Ultra Sonic Disruptor UD-211 (TOMY SEIKO Co., Ltd, Tokyo, Japan), and then centrifuged at 108,000 × *g* for 20 min at 4°C. The supernatant was loaded onto a Ni-NTA Superflow column (Qiagen) equilibrated with Buffer A linked to the BioAssist eZ system (TOSOH). The column was washed with Buffer B (50 mM sodium phosphate buffer (pH 8.0) containing 300 mM NaCl, 10% (v/v) glycerol, and 50 mM imidazole). The enzymes were then eluted with Buffer C (pH 8.0, Buffer B containing 250 mM imidazole instead of 50 mM imidazole), concentrated by ultrafiltration with Centriplus YM-30 (Millipore), dialyzed against 50 mM Tris-HCl buffer (pH 8.0) containing 50% (v/v) glycerol, and stored at −35°C until use.

The native molecular mass of recombinant proteins was estimated by gel filtration, which was carried out using a HPLC system at a flow rate of 1 ml/min. The purified enzyme was loaded onto a TSKgel G3000SWXL column (TOSOH) equilibrated with 50 mM Tris-HCl buffer (pH 8.0). A high molecular weight gel filtration calibration kit (GE Healthcare) was used for molecular markers.

### Enzyme assay by HPLC

Purified protein was added to 50 mM Tris-HCl buffer (pH 8.0) (1 ml) containing 10 mM substrate, and incubated at 50°C (for TlProR and HjProR) or 30°C (FaProR, CdProR, and AbHypE). After varying the incubation times, the enzyme reaction was stopped by rapidly incubating at −80°C. To this solution, 25 μl each of 10 mM 4-fluoro-7-nitrobenzofurazan (NBD-F) in ethanol and 100 mM borate buffer (pH 8.0) was then added and incubated at 60°C for 1 min. A total of 1.15 ml of 5 mM HCl was finally added to the reaction mixture, followed by filtration through a 0.22-μm filter (Millipore). HPLC analysis was carried out using an Ultron ES-CD chiral separation column (150 × 2.0 mm, Shinwa Chemical Industries). The flow rate was 1 ml∙min^−1^, and isocratic elution with 20 mM potassium phosphate (pH 6.0) containing 10% (v/v) methanol was monitored by absorbance at 530 nm. Non-enzymatic conversion of L↔D isomers was observed even after being incubated for 12 h, and the difference in background turnover between 30°C and 50°C was negligible. This method was used to estimate the effects of substrate specificity and inhibition by pyrrole-2-carboxylate (PYC) on activity.

### Spectrophotometric enzyme assay

Racemase/epimerase activity for proline or hydroxyproline(s) was spectrophotometrically assayed at 50°C by monitoring the reduction rate of an artificial electron acceptor in the coupling system with dehydrogenase for proline or hydroxyproline, using a Shimadzu UV-1800 spectrophotometer (Shimadzu GLC Ltd., Tokyo, Japan).

Activity toward L-proline and T3LHyp. The standard reaction mixture contained 0.05 mM 2,6-dichloroindophenol (Cl2Ind) and purified D-ProDH from *Pyrobaculum islandicum* (20 μg) [19] in 50 mM Tris-HCl buffer (pH 8.0).

Activity toward D-proline. The standard reaction mixture contained 0.05 mM Cl2Ind and purified L-ProDH from *Aeropyrum pernix* (20 μg) [20] in 50 mM Tris-HCl buffer (pH 8.0).

Activity toward T4LHyp. The standard reaction mixture contained 0.25 mM *p*-iodonitrotetrazolium violet (INT), 0.06 mM phenazine methosulfate (PMS), and purified D-HypDH from *A*. *brasilense* (20 μg) [21] in 50 mM Tris-HCl buffer (pH 8.0).

All reactions were initiated by the addition of 100 mM substrate (100 μl) with a final reaction volume of 1 ml. The millimolar absorption coefficients (ε) for Cl2Ind and INT were 19.1 mM^−1^∙cm^−1^ at 600 nm and 15.0 mM^−1^∙cm^−1^ at 490 nm, respectively. The detectable limit of specific activity was ∼0.001 unit/mg protein, due to non-enzymatic absorbance change by Cl2Ind and PMS/INT. This method was used to determine the kinetic parameters, *K*_m_ and *k*_cat_, which were calculated by a Lineweaver-Burk plot.

### Identification of reaction products

To remove glycerol in the stored buffer, purified TlProR (10 μg) was dialyzed at 4°C overnight in 50 mM potassium phosphate buffer (pH 7.0), lyophilized, and solved in D_2_O (600 μl) containing 20 mM T4LHyp or T3LHyp. NMR spectra were recorded at 25°C on a JEOL JNM-EC400 NMR spectrometer (JEOL Ltd., Tokyo, Japan) operating at 400 MHz. 2,2-Dimethyl-2-silapentane-5-sulfonate was used as an internal standard. ^1^H NMR spectra of T4LHyp and T3LHyp (400 MHz, D_2_O) were as follows: T4LHyp, 4.41 (1H, t, *J* = 4 Hz), 4.33 (1H, dd, *J*_1_ = 10 Hz, *J*_2_ = 8 Hz), 3.47 (1H, dd, *J*_1_ = 12 Hz, *J*_2_ = 4 Hz), 3.35 (1H, ddd, *J*_1_ = 12 Hz, *J*_2_ = *J*_3_ = 2 Hz), 2.42 (1H, dddd, *J*_1_ = 14 Hz, *J*_2_ = 8 Hz, *J*_3_ = *J*_4_ = 2 Hz), and 2.15 (1H, ddd, *J*_1_ = 14 Hz, *J*_2_ = 10 Hz, *J*_3_ = 4 Hz); C4DHyp, 4.57 (1H, m), 4.21 (1H, dd, *J*_1_ = 11 Hz, *J*_2_ = 4 Hz), 3.36 (1H, dd, *J*_1_ = 12 Hz, *J*_2_ = 4 Hz), 2.50 (1H, ddd, *J*_1_ = 14 Hz, *J*_2_ = 10 Hz, *J*_3_ = 4 Hz), and 2.25 (1H, m); T3LHyp, 4.68 (1H, m), 4.07 (1H, s), 3.60 (1H, m), 3.50 (1H, m), and 2.04 (2H, m); C3DHyp, 4.71 (1H, m), 4.14 (1H, d, *J* = 4 Hz), 3.56 (1H, m), 3.48 (1H, m), and 2.18 (2H, m).

### Site-directed mutagenesis

A mutation was introduced into the *TlProR*, *FaProR*, or *AbHypE* genes by sequential steps of PCR [22] using sense and antisense primers (S1 Table) and pET/TlProR_WT_ (for TlProR_F62S_ (CTG→AGG), TlProR_F62V_ (CTG→GTT), TlProR_L221H_ (CTG→GAG), TlProR_F62S/L221H_, TlProR_W241F_ (GTG→GAC), TlProR_W241C_ (CTG→TGT), and TlProR_W241Y_ mutants (CTG→TAT)), pET/FaProR_WT_ (for FaProR_F240W_ mutant (GCC→AAG)), or pET/AbHypE_WT_ (for AbHypE_C226F_ mutant (GTG→TTC)) as a template (the codons used for each mutation were in parentheses). The coding region of the mutated genes was confirmed by subsequent sequencing in both directions. Mutant proteins were expressed and purified by the same procedures as those for the wild-type enzyme.

### Amino acid sequence alignment and phylogenetic analysis

Protein sequences were analyzed using the Protein-BLAST and Clustal W program distributed by DDBJ (DNA Data Bank of Japan) (www.ddbj.nig.ac.jp). The phylogenetic tree was produced using the TreeView 1.6.1. program.

## Results

### Putative ProR or HypE, but not the T3LHypD gene from archaea

We previously reported that OCC_00387 and OCC_00362(7) from *T*. *litoralis* encoded T3LHypD and Pyr2C reductase are involved in (putative) T3LHyp metabolism, respectively [11] (Fig. 1A). These genes were clustered on the genome together with several other hypothetical genes (Fig. 1B), among which OCC_00357 (α-subunit) and OCC_00352 (β) (genes) were homologous to α_4_β_4_-type L-ProDH from *Pyrococcus horikishii* (PH1363 and PH1364 with 69 and 80% identities, respectively) [23], while OCC_00347 (α), OCC_00342 (γ), OCC_00337 (δ), and OCC_00332 (β) (genes) were similar to αβγδ-type L-ProDH from *Thermococcus profundus* (pdhA, pdhF, pdhX, and pdhB with 81, 72, 72, and 64% identities, respectively) [24]. Since T3Lhyp is metabolized to L-proline via a Pyr2C intermediate [9–11], the gene cluster may be related to both the (general) metabolism of L-proline and T3LHyp. Another ProR-like gene (OCC_00372) was also detected in this gene cluster, and the putative amino acid sequence contained two cysteine residues at the active sites (Cys^88^-Cys^251^) specific for ProR or HypE, but not T3LHypD (see below). Furthermore, by a homology search using the Protein-BLAST program, some archaea including *F*. *acidarmanus* (*F*. *acidiphilum*) and *H*. *japonica* were found to possess the homologous gene (see below in detail). Therefore, in the present study, we selected three ProR-like genes from *T*. *litoralis*, *F*. *acidiphilum*, and *H*. *japonica* as target genes (referred to as TlProR, FaProR, and HjProR genes, respectively). Furthermore, ProR from *C*. *difficile* [13] and HypE from *A*. *brasilense* [21] were used as controls (referred to as CdProR and AbHypE, respectively).

### Preparation of recombinant His_6_-tag proteins

After cloning all target genes into the vector pETDuet-1, the recombinant enzymes were successfully expressed in *E*. *coli* cells as His_6_-tagged enzymes, and purified to homogeneity using a nickel-chelating affinity column (Fig. 1C). Additional amino acid residues including the His_6_-tag at the N-terminal was confirmed by western blot analysis with an anti-His_6_-tag antibody (Fig. 1D). The apparent molecular masses of TlProR, FaProR, HjProR, CdProR, and AbHypE, as estimated by SDS-PAGE, were 40 (38.1), 40 (38.5), 42 (35.8), 40 (37.7) and 37 (34.1) kDa (values in parentheses indicate the calculated molecular mass of the enzyme with His_6_-tag), while those estimated by analytic gel filtration were ∼80 kDa, respectively (data not shown). Therefore, all these enzymes appeared to be dimeric.

### Substrate specificity of TlProR

Stereoisomer identification and quantification by potential enzyme reactions were determined by chiral separation column chromatography, in which a sample was derivatized with NBD-F. The typical retention times (min) of the tested substrates (Fig. 2) were follows: L-proline, 12.9; D-proline, 11.6; T4LHyp, 8.2; C4DHyp, 8.7; C4LHyp, 9.5; T4DHyp, 7.1; T3LHyp, 8.0; (putative) C3DHyp, 8.5; C3LHyp, 9.8; (putative) T3DHyp, 7.3; L-pipecolate, 15.4; D-pipecolate, 14.7; L-azetidine-2-carboxylate, 9.1; (putative) D-azetidine-2-carboxylate, 8.6.

**Fig 3:**
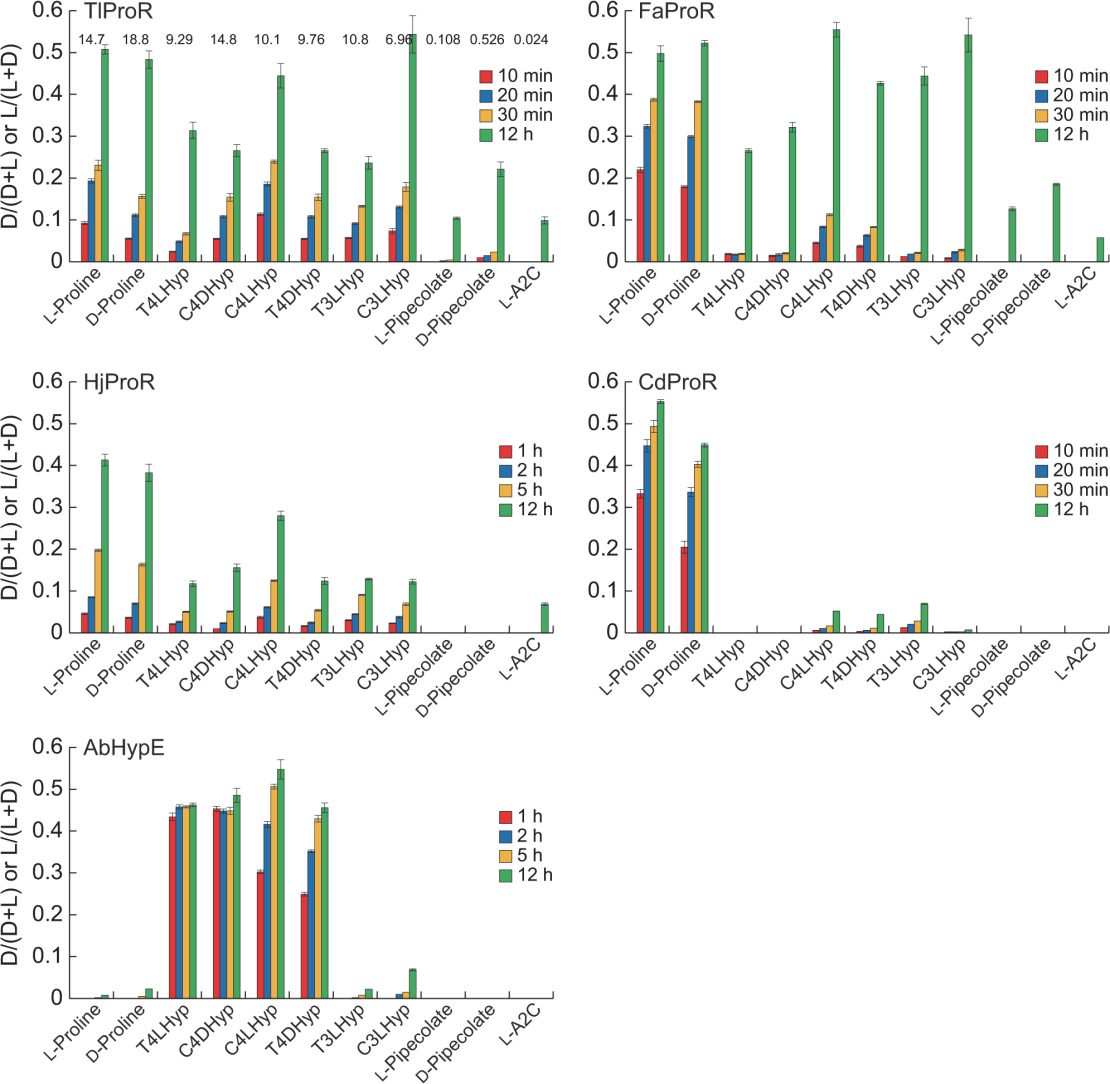
Substrate specificities of TlProR, FaProR, HjProR, CdProR, and AbHypE. The reaction mixture (1 ml) consisted of 10 mM of substrate and each purified enzyme as follows in Tris-HCl buffer (pH 8.0): TlProR, 1.3 μg; FaProR, 100 μg; HjProR, 570 μg; CdProR, 3.6 μg; AbHypE, 4.9 μg. L-A2C is L-azetidine-2-carboxylate. Samples at the indicated times were analyzed by HPLC (means ± S.D., *n* = 3). Values on the bars of TlProR indicate specific activity (unit/mg protein), which are similar to those in Tables 1 and 2.

TlProR could catalyze the conversion of not only L-proline, but also 4-hydroxy-L-proline (T4LHyp (63) and C4LHyp (69)) and 3-hydroxy-L-proline (T3LHyp (73) and C3LHyp (47)) (values in parentheses indicate specific activity as a percentage of L-proline) (Fig. 3). The reaction was completely bi-directional, and the reverse reactions were also similar: D-proline (130), C4DHyp (160), and T4DHyp (97) (values in parentheses indicate specific activity as a percentage of L-enantiomer). Furthermore, using large amounts of the purified enzyme, we could also estimate specific activities for L-pipecolate (0.7), D-pipecolate (3.6), and L-azetidine-2-carboxylate (0.16), which are the six- and four-membered ring analogs of proline, respectively.

**Table 1:** Kinetic parameters for L-proline, T4LHyp, and T3LHyp.

Enzymes	Substrates	Specific activity ^a^ (units/mg protein)	*K*_m_ (mM)	*k*_cat_ (min^−1^)	*k*_cat_/*K*_m_ (min^−1^·mM^−1^)	$\frac{ProR}{HypE}$
TlProR_WT_	L-Proline	3.45	1.21±0.12	167±10	138±4	‒
	T4LHyp	9.69	6.08±1.76	728±146	122±13	1.1 ^b^
	T3LHyp	3.67	0.502±0.021	216±4	430±10	0.32
TlProR_W241F_	L-Proline	5.19	1.29±0.14	190±14	147±5	‒
	T4LHyp	2.13	4.28±0.84	92.0±10.5	21.7±1.7	6.8
	T3LHyp	0.160	16.4±3.2	17.5±2.5	1.07±0.07	137
TlProR_L221H_	L-Proline	0.488	20.5±2.5	55.4±6.1	2.71±0.05	0.45
	T4LHyp	0.670	6.50±0.39	39.0±1.7	6.01±0.11	
TlProR_F62S_	L-Proline	0.0195	154±13	12.0±1.1	0.0774±0.0010	0.20
	T4LHyp	0.0384	6.22±0.62	2.35±0.17	0.379±0.012	
TlProR_F62V_	L-Proline	0.0691	1.93±0.44	5.51±0.99	2.88±0.15	0.33
	T4LHyp	0.0972	0.905±0.075	7.84±0.49	8.67±0.18	
TlProR_F62S/L221H_	L-Proline	0.001>	N.D. ^c^	N.D.	N.D.	‒
	T4LHyp	0.001>	N.D.	N.D.	N.D.	
FaProR_WT_	L-Proline	0.29	31.6±5.3	35.8±5.6	1.13±0.01	2.9
	T4LHyp	0.0209	6.12±0.30	2.37±0.10	0.387±0.002	
FaProR_F240W_	L-Proline	0.117	53.0±8.0	25.0±3.4	0.473±0.008	1.4
	T4LHyp	0.00822	2.17±0.13	0.717±0.032	0.331±0.005	
CdProR	L-Proline	14.2	8.53±0.47	849±27	99.6±57	‒
	T4LHyp	0.0975	N.D.	N.D.	N.D.	
AbHypE_WT_	L-Proline	0.124	N.D.	N.D.	N.D.	‒
	T4LHyp	58.6	1.39±0.15	4400±340	3180±100	
AbHypE_C226F_	L-Proline	0.001>	N.D.	N.D.	N.D.	‒
	T4LHyp	0.001>	N.D.	N.D.	N.D.	

^a^Under standard assay conditions in the “Materials and Methods”.

^b^Ratio of L-proline to T4LHyp or T3LHyp in *k*_cat_/*K*_m_.

^c^Not determined due to trace activity.

**Table 2:** Kinetic parameters for D-proline and C4DHyp.

Enzymes	Substrates	Specific activity ^a^ (units/mg protein)	*K*_m_ (mM)	*k*_cat_ (min^−1^)	*k*_cat_/*K*_m_ (min^−1^·mM^−1^)	$\frac{ProR}{HypE}$
TlProR_WT_	D-Proline	3.45	0.0919±0.006	133±2	1570±90	45.1 ^b^
	C4DHyp	3.48	6.65±0.69	230±12	34.8±1.8	
TlProR_W241F_	D-Proline	4.87	0.194±0.020	191±5	990±82	158
	C4DHyp	0.681	6.70±0.68	41.8±2.7	6.25±0.24	
TlProR_L221H_	D-Proline	0.299	2.54±0.46	17.3±2.7	6.84±0.19	4.3
	C4DHyp	0.111	4.18±0.81	6.66±1.31	1.59±0.00	
TlProR_F62S_	D-Proline	0.0283	4.00±0.17	1.55±0.09	0.387±0.008	1.4
	C4DHyp	0.0186	3.14±0.15	0.859±0.012	0.274±0.010	
TlProR_F62V_	D-Proline	0.0309	0.156±0.004	1.41±0.03	9.04±0.23	‒
	C4DHyp	0.00661	N.D.	N.D.	N.D.	
TlProR_F62S/L221H_	D-Proline	>0.001	N.D.	N.D.	N.D.	‒
	C4DHyp	>0.001	N.D.	N.D.	N.D.	
FaProR_WT_	D-Proline	0.164	1.17±0.09	7.09±0.12	6.09±0.35	‒
	C4DHyp	0.00234	N.D. ^c^	N.D.	N.D.	
FaProR_F240W_	D-Proline	0.141	3.12±0.08	7.21±0.06	2.31±0.04	‒
	C4DHyp	0.00230	N.D.	N.D.	N.D.	
CdProR	D-Proline	3.92	0.612±0.158	149±34	245±8	‒
	C4DHyp	>0.001	N.D.	N.D.	N.D.	

^a^Under standard assay conditions in the “Materials and Methods”.

^b^Ratio of D-proline to C4DHyp in *k*_cat_/*K*_m_.

^c^Not determined due to trace activity.

**Fig 4:**
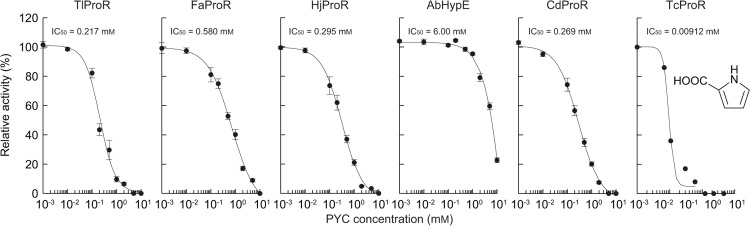
Unique inhibition of archaeal ProR-like enzymes by pyrrole-2-carboxylate (PYC; inset). Reactions were performed for 30 min with the same conditions as those in Fig. 3, except for the presence of several concentration of PYC. L-Proline (for TlProR, FaProR, HjProR, and CdProR) or T4LHyp (for AbHypE) was used as a substrate. Relative specific activity values were expressed as percentages of the values obtained in the absence of PYC (means ± S.D., *n* = 3). Data for the ProR of *T*. *cruzi* (TcProR) are from Berneman et al. [25]. IC_50_ values were calculated by curve fitting using ImageJ software (http://rsb.info.nih.gov/ij/).

Another previously reported difference in the properties of ProR and HypE is the profile of inhibition by pyrrole-2-carboxylate (PYC), a transition state analogue of proline [6, 13, 25]: ProR enzymes from trypanosomatids and clostridia were inhibited at even 0.01 and 1 mM, respectively, whereas HypE reactions were only affected by high amounts of PYC (∼10 mM), as described in CdProR and AbHypE (Fig. 4). In spite of the significant HypE activity, the IC_50_ value of TlProR (0.217 mM) was ∼28-fold higher than that of AbHypE (6.00 mM), confirming dual activity toward proline and 4-hydroxyproline.

### Kinetic analysis of TlProR

A more sensitive spectrophotometric assay method using proline or hydroxyproline dehydrogenase as a coupling enzyme was developed to estimate kinetic properties. The kinetic parameters of proline and hydroxyproline determined from the Lineweaver-Burk plot are shown in Tables 1 (L→D) and 2 (D→L). The catalytic efficiency (*k*_cat_/*K*_m_) values for L-proline and T4LHyp were similar, whereas a preference for D-proline over C4DHyp (45-fold) was identified and was caused by a 75-fold lower *K*_m_ for D-proline. This may have been partially due to differences in the *K*_m_ values of L-ProDH (0.28 mM) [20] and D-ProDH (4.2 mM) [19] as the coupling enzyme used. TlProR was assayed at 50°C, which was far from the optimum temperature (90∼100°C; data not shown). Although the *k*_cat_/*K*_m_ value for T4LHyp of TlProR was 27-fold lower than that for AbHypE, the specific activity of the former at 100°C, estimated by HPLC analysis, was similar to that of the latter: 29.1 and 58.6 unit/mg protein, respectively.

Zhao et al. [26] recently characterized 51 ProR-like proteins from bacteria. Of these, the *k*_cat_/*K*_m_ value for T4LHyp of HypE from *Pseudomonas putida* was 192 and 3430-fold higher than those for T3LHyp and L-proline, respectively, and was caused by a markedly higher *k*_cat_ value. Similar results were also obtained for other (putative) HypE proteins. In contrast, the kinetic constant for T3LHyp of TlProR was similar to those of L-proline and T4LHyp. Thus, to the best of our knowledge, this is the first example of a ProR-like protein being capable of utilizing not only proline, but also 4-hydroxyproline and 3-hydroxyproline as a substrate.

### Identification of reaction products by TlProR

**Fig 5:**
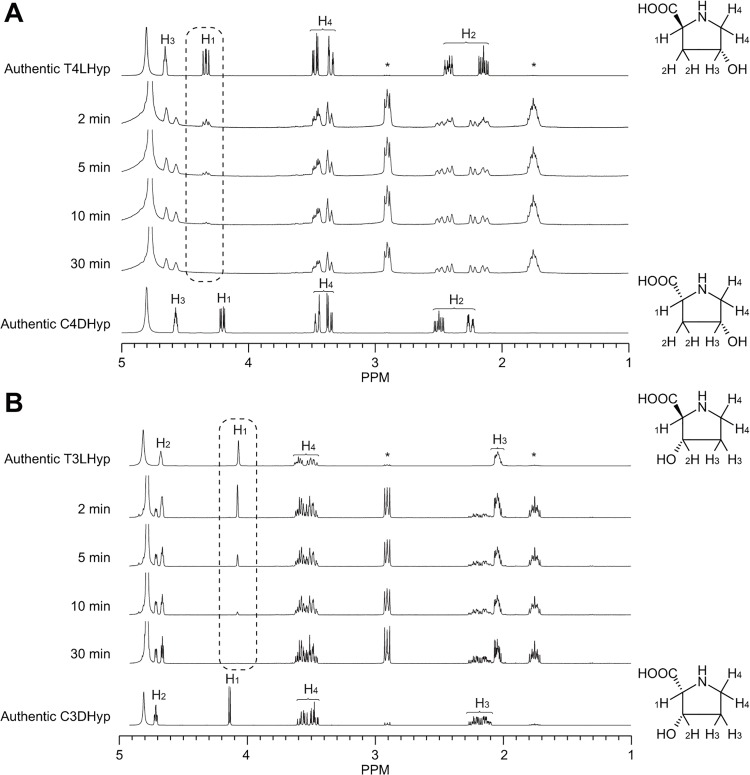
Representative ^1^H NMR spectra for epimerase activity toward T4LHyp (A) and T3LHyp (B) by TlProR. Asterisks are peaks derived from an internal standard. Left panels show the assignments of protons in D_2_O. The dashed line box indicates the progressive loss of H_1_ peaks.

T4LHyp was incubated with TlProR in D_2_O at various incubation times. ^1^H NMR spectra showed the progressive loss of H_1_ peaks, and additionally contained resonances associated with C4DHyp, a potential product: an exchange of the α-proton with solvent deuterium (Fig. 5A). Similar phenomena were also observed when T3LHyp was used as a substrate instead of T4LHyp (Fig. 5B). These results were expected for a 1,1-proton transfer reaction that equilibrates the configurations at Cα of not only 4-hydroxyproline, but also 3-hydroxyproline (novel activity), as proposed previously [12].

### Phylogenetic analysis of TlProR

**Fig 6:**
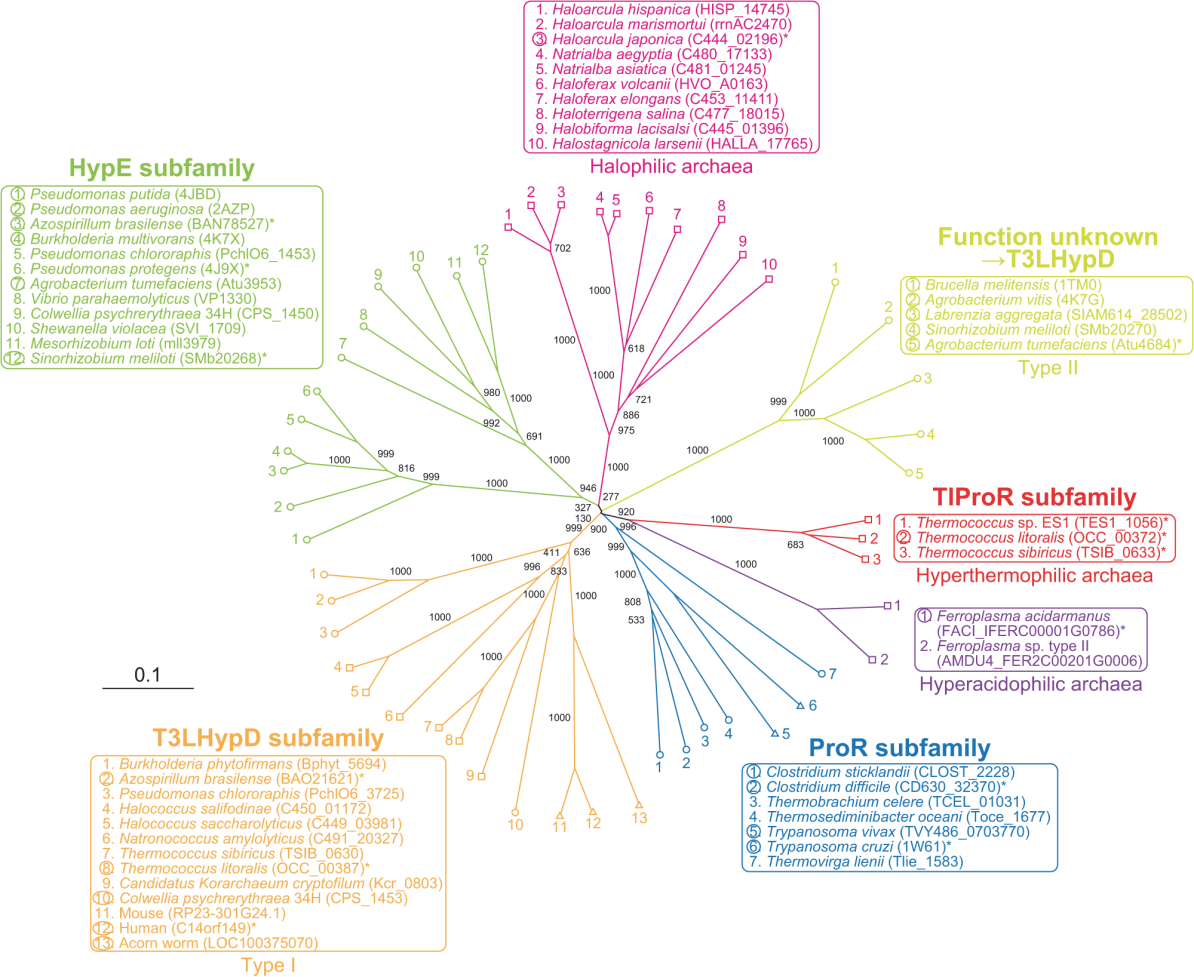
Phylogenetic tree of the ProR superfamily. The number on each branch indicates the bootstrap value. The circles, squares, and triangles at the end of each branch are enzymes from bacteria, archaea, and eukaryotes, respectively. Proteins with asterisks were used for Fig. 7. Proteins in circles were functionally characterized.

As expected from the preliminary annotation, TlProR belongs to the ProR superfamily, which consists of four subfamilies: the archetype ProR (blue in Fig. 6), HypE (light green), T3LHypD (orange), and function unknown protein (yellow). Only two putative proteins from the hyperthermophilic archaeon *Thermococcus sibiricus* (TSIB_0633) and *Thermococcus* sp. ES1 (TES1_1056) showed >85% sequential identity (referred to as TlProR subfamily; red), and the corresponding genes formed a similar gene cluster to *T*. *litoralis*, together with (putative) L-ProDH and Pyr2C reductase genes (Fig. 1B). Although TlProR (subfamily) is not strongly related to any of the subclasses of the other members (30∼40% sequence identity), a Protein-BLAST analysis revealed that TlProR was a close, but distinct subfamily of the ProR subfamily; the (putative) ligand binding sites were similar to those of ProR, but not HypE or T3LHypD, as described below. Furthermore, we found that two putative proteins (Cys-Cys type) from the hyperacidophilic archaeon *F*. *acidarmanus* (FaProR) and *Ferroplasma* sp. type II were located between the TlProR and ProR subfamilies (purple in Fig. 6). Several halophilic archaea including *H*. *japonica* (HjProR) (pink) also possessed similar types of proteins (genes), but formed a distinct subfamily to any other subfamily, and were often clustered with putative bacterial type (but not archaeal) L-ProDH (PutA) (Fig. 1B).

### Characterization of FaProR and HjProR

To further estimate the evolutionary insight of archaeal ProR-like protein(s), FaProR and HjProR were characterized enzymatically using purified recombinant proteins (Fig. 1C). The racemization activity of proline was found to be significant in FaProR, although specific activity was ∼10-fold lower than that of TlProR (Fig. 3). On the other hand, the *k*_cat_/*K*_m_ value for T4LHyp was ∼3-fold lower than that for L-proline, which was attributed to a 17-fold lower *k*_cat_ value, and we could not determine the kinetic parameters of the epimerization of C4DHyp to T4DHyp due to this low activity (Tables 1 and 2). Although similar results were obtained from the HPLC analysis (Fig. 3), L- or D-hydroxyproline(s) was significantly converted to another enantiomer(s) after being incubated for 12 h. These results suggested that the substrate specificity of FaProR was more similar to the known ProR enzymes than that of TlProR, confirming a phylogenetic relationship, as described above.

Although HjProR could also catalyze proline racemization, (low) specific activity was unmeasurable possibly due to the partial proteolysis (Fig. 1CD) and/or the enzyme from extreme halophilus: activation by salts is frequency necessary for full activity recovery [27]. Therefore, substrate specificity was preliminarily estimated by HPLC analysis using samples that had been incubated for 12 h (Fig. 3), and the enzyme could utilize both proline and hydroxyproline(s) as a substrate, similar to TlProR.

### Identification of amino acid residue(s) for discriminating between proline and hydroxyproline

**Fig 7:**
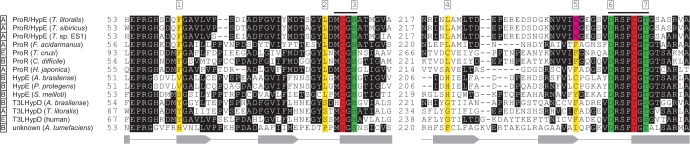
Partial multiple sequence alignment of deduced amino acid sequences of TlProR. A, B, and E are enzymes from archaea, bacteria, and eukaryotes, respectively. Consensus segments of the ProR superfamily are shown as a line on the sequence. Catalytic cysteine and/or threonine residues are shaded in red. Gray-shaded letters indicate highly conserved amino acid residues. Seven substrate binding sites are shaded in yellow and green, and the former interact with the carboxyl group or pyrrolidine nitrogen atom. The tryptophan residue, which is important for the discrimination of substrates (Typ^241^ in TlProR), is shaded in pink. Gray-shaded letters indicate highly conserved amino acid residues. The secondary structures of ProR from *T*. *cruzi* (PDB ID 1W61), α-helix (rectangles) and β-sheets (arrows), are shown under the sequence.

In the crystal structure of the enzyme-T4LHyp complex of HypE from *Pseudomonas protegens* (PDB ID 4J9X) (33% identity with TlProR) [26], seven amino acid residues were detected close to the ligands, except for two catalytic cysteine residues (referred to as sites 1∼7) (Fig. 7). Among them, sites 3, 6, and 7 were previously reported to be contained in two conserved motifs for the ProR superfamily (Met^87^-Cys-Gly-His ^90^ and Asp ^247^-Arg-Ser-Pro-Cys-Gly-Thr-Gly^254^; the numbers for TlProR), and formed hydrogen bonds with the carboxyl group or pyrrolidine nitrogen atom (shaded in green) [13].

TlProR possessed a tryptophan residue (Trp^241^) at site 5 (shaded in pink), which was substituted to phenylalanine or cysteine/tyrosine in all known ProR (and also FaProR and HjProR) and HypE enzymes, respectively. Therefore, we constructed TlProR_W241F_, TlProR_W241C_, TlProR_W241Y_, FaProR_F241W_, and AbHypE_C226F_ mutants at an equivalent position. Of these, only the TlProR_W241F_, FaProR_F240W_, and AbHypE_C226F_ mutants successfully expressed in *E*. *coli* cells, as well as the wild-type (WT) enzyme (Fig. 1C). TlProR_W241F_ showed 5.3, 430, and 5.8-fold lower *k*_cat_/*K*_m_ values for T4LHyp, T3LHyp, and C4DHyp respectively, mainly due to a marked decrease in *k*_cat_ values, whereas no significant effects were found in the kinetic constants of proline (Tables 1 and 2). FaProR_F241W_ showed similar kinetic constants for proline and hydroxyproline to the WT enzyme. The AbHypE_C226F_ mutant could utilize neither proline nor hydroxyproline(s) as a substrate. These results clearly indicated that tryptophan in the TlProR residue played important role(s) in both substrate specificity and structural folding, and favorably interacted with both the 4 and 3-hydroxy groups of hydroxyproline.

Phenylalanine at site 1 for ligand binding may only exist in ProR(-like) enzymes including TlProR, while histidine at site 4 could be specific for HypE. The three constructed TlProR mutants, F62S, F62V, and L221H, which mimic the natural HypE enzyme, moderately shifted substrate specificity toward 4-hydroxyproline (in particular, direction of L→D), and this was mainly attributed to a marked decrease in *k*_cat_ values; however, the prominent loss of both ProR and HypE catalysis was also observed, and there was no synergistic effect in a double mutant, F62S/L221H (Tables 1 and 2).

## Discussion

The most interesting finding of the present study was the manner by which TlProR recognized both proline and 4-hydroxyproline (and 3-hydroxyproline) as substrates. Goytia et al. [13] proposed that several amino acid residues were responsible for this discrimination, in which two aromatic phenylalanine residues of ProR (at sites 1 and 5 in Fig. 7) may play the most important role(s) through hydrophobic interaction(s) between the enzyme and pyrrolidine ring of proline. If this hypothesis is correct, a mutation at the corresponding sites in ProR or HypE could significantly change each substrate specificity. However, when these amino acid residues are exchanged between ProR and HypE, the resultant mutant(s) can generally utilize neither proline nor hydroxyproline as a substrate: V60F for HypE from *Pseudomonas aeruginosa* [13], F62S and F62V for TlProR (this study), and C226F for AbHypE (this study). On the other hand, there is no doubt that TlProR (directly) evolved from an ancestor with phenylalanine at site 5 such as FaProR (Fig. 6). In other words, the W241F mutation (but not W241C and W241Y) mimicked the natural evolutionary process, through which no radical loss of activity occurred.

FaProR possessed the same ligand binding sites as natural ProR (Fig. 7). Therefore, it is unexpected that the (hydrophobic and bulky) tryptophan at site 5 of TlProR played an important role in the activity of hydroxyproline, which is more hydrophilic and bulkier than proline. No equivalent tryptophan was detected in the other members of the ProR superfamily, and many HypE enzymes including *Sinorhizobium meliloti* [28] possessed (the same aromatic and hydrophilic) tyrosine residue at that position, strongly suggesting that substrate specificity had been convergently acquired between TlProR and (natural) HypE. On the other hand, the environment in the active site pocket of TlProR seems to be suitable for recognizing hydroxyproline as a substrate independently of the amino acid residue at site 5, because FaProR, which possesses phenylalanine, but not tryptophan, at this position, as well as TlProR, shows similar IC_50_ value for inhibition by PYC to TlProR (Fig. 4). Several bacterial ProR-like proteins of the Ser-Thr type at the (putative) active sites (see in “Introduction”) have recently been functionally annotated as T3LHypD (Type II in Fig. 6) [26]. The ligand binding sites (1, 2, 4, and 5) were completely different from those of known T3LHypD (Type I) (Fig. 7). These results indicate that convergent evolution frequently occurred in the ProR superfamily, and also that there was more than one pattern for favorable binding of the same substrate. A previous study proposed that the last universal common ancestor (well denoted “LUCA” or “LCA”) was a hyperthermophilic organism [29] such as *T*. *litoralis* (98°C of maximum temperature for the growth) [15], and also that they possessed enzymes with significant broad substrate specificity [30]; therefore, the unique properties of TlProR may be (partially) derived from an ancient protein of the ProR superfamily.

The physiological role of TlProR has not yet been established. Since *T*. *litoralis* possesses no homologous gene to D-proline reductase, the physiological function of TlProR may be to epimerize hydroxyproline, but not racemize proline, confirming the evolutionary scenario proposed above. *T*. *litoralis* significantly accumulated T4LHyp as an intercellular organic solute, while other *Thermococcus* species including *T*. *celer*, *T*. *stetteri*, and *T*. *zilligii* did not [31]. Furthermore, archaeal D-ProDH was previously shown to be capable of converting C4DHyp to Pyr4H2C [19]; therefore, the homologous pathway involved in the metabolism of T4LHyp by bacteria may also be operative in *T*. *litoralis* [7, 8]. In another hypothesis, Pyr5C was suggested to be spontaneously taumerized to L-glutamate γ-semialdehyde via a “5-hydroxy-L-proline” intermediate (Fig. 1A). If TlProR is also able to epimerize 5-hydroxy-L-proline to the D-enantiomer due to broad substrate specificity, D-glutamate is finally produced via D-glutamate γ-semialdehyde. In bacteria, D-glutamate was reported to be produced from L-glutamate by glutamate racemase (EC 5.1.1.3), and used as a building block of cell-wall peptidoglycan [32]. Although D-glutamate has also been detected in several archaea, the homolog from archaea functions as an aspartate racemase (EC 5.1.1.13) [33]. Therefore, TlProR may be involved in an alternative pathway of D-glutamate biosynthesis. The development of the gene disruption of *T*. *litoralis* will be useful for understanding the physiological role(s) of the *TlProR* gene in more detail.

## Supporting Information

S1 Table Primers used in this study. ^a^Lower case letters indicate additional bases for introducing the digestion sites of restriction enzymes in parentheses. ^b^Only sense primers are shown. Underlining indicates mutated regions.
(PDF)

## References

[1] Wu G, Bazer FW, Burghardt RC, Johnson GA, Kim SW, Knabe DA, et al Proline and hydroxyproline metabolism: implications for animal and human nutrition. Amino Acids. 2011;40: 1053–1063. 10.1007/s00726-010-0715-z 20697752 PMC3773366

[2] Bouillaut L, Self WT, Sonenshein AL. Proline-dependent regulation of *Clostridium difficile* Stickland metabolism. J Bacteriol. 2013;195: 844–854. 10.1128/JB.01492-12 23222730 PMC3562115

[3] Jackson S, Calos M, Myers A, Self WT. Analysis of proline reduction in the nosocomial pathogen *Clostridium difficile* . J Bacteriol. 2006;188: 8487–8495. 17041035 10.1128/JB.01370-06 PMC1698225

[4] Chamond N, Grégoire C, Coatnoan N, Rougeot C, Freitas-Junior LH, da Silveira JF, et al Biochemical characterization of proline racemases from the human protozoan parasite *Trypanosoma cruzi* and definition of putative protein signatures. J Biol Chem. 2003;278: 15484–15494. 12735293 10.1074/jbc.m210830200

[5] Reina-San-Martín B, Degrave W, Rougeot C, Cosson A, Chamond N, Cordeiro-Da-Silva A, et al A B-cell mitogen from a pathogenic trypanosome is a eukaryotic proline racemase. Nat Med. 2000;6: 890–897. 10932226 10.1038/78651

[6] Chamond N, Cosson A, Coatnoan N, Minoprio P. Proline racemases are conserved mitogens: characterization of a *Trypanosoma vivax* proline racemase. 2009;Mol Biochem Parasitol. 165: 170–179. 10.1016/j.molbiopara.2009.02.002 19428664

[7] Watanabe S, Yamada M, Ohtsu I, Makino K. α-Ketoglutaric semialdehyde dehydrogenase isozymes involved in metabolic pathways of D-glucarate, D-galactarate and hydroxy-L-proline: molecular and metabolic convergent evolution. J Biol Chem. 2007;282: 6685–6695. 17202142 10.1074/jbc.M611057200

[8] Watanabe S, Morimoto D, Fukumori F, Shinomiya H, Nishiwaki H, Kawano-Kawada M, et al Identification and characterization of D-hydroxyproline dehydrogenase and Δ^1^-pyrroline-4-hydroxy-2-carboxylate deaminase involved in novel L-hydroxyproline metabolism of bacteria: metabolic convergent evolution. J Biol Chem. 2012;287: 32674–32688. 22833679 10.1074/jbc.M112.374272 PMC3463351

[9] Visser WF, Verhoeven-Duif NM, de Koning TJ. Identification of a human *trans*-3-hydroxy-L-proline dehydratase, the first characterized member of a novel family of proline racemase-like enzymes. J Biol Chem. 2012;287: 21654–21662. 10.1074/jbc.M112.363218 22528483 PMC3381129

[10] Watanabe S, Tanimoto Y, Yamauchi S, Tozawa Y, Sawayama S, Watanabe Y. Identification and characterization of *trans*-3-hydroxy-L-proline dehydratase and Δ^1^-pyrroline-2-carboxylate reductase involved in *trans*-3-hydroxy-L-proline metabolism of bacteria. FEBS Open Bio. 2014;4: 240–250. 10.1016/j.fob.2014.02.010 24649405 PMC3958920

[11] Watanabe S, Tozawa Y, Watanabe Y. Ornithine cyclodeaminase/μ-crystallin homolog from hyperthermophilic archaeon *Thermococcus litoralis* functions as a novel Δ^1^-pyrroline-2-carboxylate reductase involved in putative *trans*-3-hydroxy-L-proline metabolism. FEBS Open Bio. 2014;4: 617–626. 10.1016/j.fob.2014.07.005 25161870 PMC4141209

[12] Buschiazzo A, Goytia M, Schaeffer F, Degrave W, Shepard W, Grégoire C, et al Crystal structure, catalytic mechanism, and mitogenic properties of *Trypanosoma cruzi* proline racemase. Proc Natl Acad Sci U S A. 2006;103: 1705–1710. 16446443 10.1073/pnas.0509010103 PMC1413642

[13] Goytia M, Chamond N, Cosson A, Coatnoan N, Hermant D, Berneman A, et al Molecular and structural discrimination of proline racemase and hydroxyproline-2-epimerase from nosocomial and bacterial pathogens. PLoS One. 2007;2: e885 17849014 10.1371/journal.pone.0000885 PMC1964878

[14] Finlay TH, Adams E. Kinetic and structural studies of hydroxyproline 2-epimerase. J Biol Chem. 1970;245: 5248–5260. 5469165

[15] Neuner A, Jannasch HW, Belkin S, Stetter KO. *Thermococcus litoralis* sp. nov.: a new species of extremely thermophilic marine archaebacteria Arch Microbiol. 1990;153: 205–207.

[16] Sambrook J, Fritsch EF, Maniatis T. Molecular Cloning: a Laboratory Manual, 3rd ed Cold Spring Harbor, NY: Cold Spring Harbor Laboratory; 2001.

[17] Lowry OH, Rosebrough NJ, Farr AL, Randall RJ. Protein measurement with the folin phenol reagent. J Biol Chem. 1951;193: 265–275. 14907713

[18] Laemmli UK. Cleavage of structural proteins during the assembly of the head of bacteriophage T4. Nature. 1970;227: 680–685. 5432063 10.1038/227680a0

[19] Satomura T, Kawakami R, Sakuraba H, Ohshima T. Dye-linked D-proline dehydrogenase from hyperthermophilic archaeon *Pyrobaculum islandicum* is a novel FAD-dependent amino acid dehydrogenase. J Biol Chem. 2002;277: 12861–12867. 11823469 10.1074/jbc.M112272200

[20] Sakuraba H, Satomura T, Kawakami R, Kim K, Hara Y, Yoneda K, et al Crystal structure of novel dye-linked L-proline dehydrogenase from hyperthermophilic archaeon *Aeropyrum pernix* . J Biol Chem. 2012;287: 20070–20080. 10.1074/jbc.M111.319038 22511758 PMC3370190

[21] Watanabe S, Hiraoka Y, Endo S, Tanimoto Y, Tozawa Y, Watanabe Y. An enzymatic method to estimate the content of L-hydroxyproline. J Biotechnol. 2015 10.1016/j.jbiotec.2015.01.026 25678137

[22] Penning TM, Jez JM. Enzyme redesign. Chem Rev. 2001;101: 3027–3046. 11710061 10.1021/cr000049n

[23] Kawakami R, Sakuraba H, Tsuge H, Goda S, Katunuma N, Ohshima T. A second novel dye-linked L-proline dehydrogenase complex is present in the hyperthermophilic archaeon *Pyrococcus horikoshii* OT-3. FEBS J. 2005;272: 4044–4054. 16098188 10.1111/j.1742-4658.2005.04810.x

[24] Kawakami R, Sakuraba H, Ohshima T. Gene and primary structures of dye-linked L-proline dehydrogenase from the hyperthermophilic archaeon *Thermococcus profundus* show the presence of a novel heterotetrameric amino acid dehydrogenase complex. Extremophiles. 2004;8: 99–108. 15064976 10.1007/s00792-003-0368-x

[25] Berneman A, Alves-Ferreira M, Coatnoan N, Chamond N, Minoprio P. Medium/high throughput D-amino acid oxidase colorimetric method for determination of D-amino acids. application for amino acid racemases. J Microbial Biochem Technol. 2010;2: 139–146.

[26] Zhao S, Sakai A, Zhang X, Vetting MW, Kumar R, Hillerich B, et al Prediction and characterization of enzymatic activities guided by sequence similarity and genome neighborhood networks. Elife. 2014;3: e03275. 10.7554/eLife.03275 PMC4113996 24980702

[27] Madern D, Ebel C, Zaccai G. Halophilic adaptation of enzymes. Extremophiles. 2000;4: 91–98. 10805563 10.1007/s007920050142

[28] White CE, Gavina JM, Morton R, Britz-McKibbin P, Finan TM. Control of hydroxyproline catabolism in *Sinorhizobium meliloti* . Mol Microbiol. 2012;85: 1133–1147. 10.1111/j.1365-2958.2012.08164.x 22804907

[29] Akanuma S, Nakajima Y, Yokobori S, Kimura M, Nemoto N, Mase T, et al Experimental evidence for the thermophilicity of ancestral life. Proc Natl Acad Sci U S A. 2013;110: 11067–11072. 10.1073/pnas.1308215110 23776221 PMC3703998

[30] Jensen RA. Enzyme recruitment in evolution of new function. Annu Rev Microbiol. 1976;30: 409–425. 791073 10.1146/annurev.mi.30.100176.002205

[31] Lamosa P, Martins LO, Da Costa MS, Santos H. Effects of temperature, salinity, and medium composition on compatible solute accumulation by *Thermococcus* spp. Appl Environ Microbiol. 1998;64: 3591–3598. 9758772 10.1128/aem.64.10.3591-3598.1998 PMC106469

[32] Fisher SL. Glutamate racemase as a target for drug discovery. Microb Biotechnol. 2008;1: 345–360. 10.1111/j.1751-7915.2008.00031.x 21261855 PMC3815242

[33] Long Z, Lee JA, Okamoto T, Sekine M, Nimura N, Imai K, et al Occurrence of D-amino acids and a pyridoxal 5’-phosphate-dependent aspartate racemase in the acidothermophilic archaeon, *Thermoplasma acidophilum* . Biochem Biophys Res Commun. 2001;281: 317–321. 11181048 10.1006/bbrc.2001.4353

