# Supplementary material for: Identification and Characterization of Bifunctional Proline Racemase/Hydroxyproline Epimerase from Archaea: Discrimination of Substrates and Molecular Evolution
Source: PLoS One. 2015 Mar 18;10(3):e0120349. doi: 10.1371/journal.pone.0120349 (PMC4364671; doi:10.1371/journal.pone.0120349)
Supplement: S1 Table — aLower case letters indicate additional bases for introducing the digestion sites of restriction enzymes in parentheses. bOnly sense primers are shown. Underlining indicates mutated regions. (PDF) [file pone.0120349.s001.pdf]

S1 Table. Primers used in this study.

Primers [a]		Sequences [a]
Cloning of the *TlProR* gene		
P1 (BglII)		5′-catagatctgTTTGCAGATCATGTCTTCCATGTTGTGG-3′
P2 (SalI)		5′-tgggtcgacTTACCTTAACAAGAAGCCCTTCCAAAG-3′
Cloning of the *HjProR* gene		
P3 (BamHI)		5′-catggatccgGAACGAACGGAGTTTACCACGGTAGAC-3′
P4 (PstI)		5′-tggctgcagTCAAGCGAGAGTGAAACTGCCGAGCG-3′
Cloning of the *FaProR* gene		
P5 (BglII)		5′-catagatctgAGCTTGAAATCTGATTTTACTATTGATG-3′
P6 (SalI)		5′-tgggtcgacTCACTGTACCAGAAACCCGTGTTTTAAAG-3′
Cloning of the *CdProR* gene		
P7 (BamHI)		5′-catggatccgAAATTTAGCAGAAGTATACAAGCTATAG-3′
P8 (SalI)		5′-tgggtcgacTTATTTAAGAATAAATCCATGTTTAAG-3′
Cloning of the *AbHypE* gene		
P9 (BamHI)		5′-catggatccgAAGCGCATCCAGATCATCGATTCGC-3′
P10 (HindIII)		5′-tggctcgagTCACGGCGCAATACCCCAGCCGAATGG-3′
Site-directed mutagenesis [b]		
P11 (TlProR$_{W241F}$)		5′-CGTCGTAATATttGGAGAGGGAAGC-3′
P12 (TlProR$_{W241Y}$)		5′-CGTCGTAATATatGGAGAGGGAAGC-3′
P13 (TlProR$_{W241C}$)		5′-CGTCGTAATATGtGGAGAGGGAAGC-3′
P14 (TlProR$_{L221H}$)		5′-GAATAGAATAAATCaCGCAATGCTCAC-3′
P15 (TlProR$_{F62S}$)		5′-CATTCGGATCAATcTGGGGCAGTTC-3′
P16 (TlProR$_{F62V}$)		5′-CATTCGGATCAAgTTGGGGCAGTTC-3′
P17 (AbHypE$_{C226F}$)		5′-GCTTCGTGCTGTtCCCGGGCCTCGC-3′
P18 (FaProR$_{F240W}$)		5′-CAATAGTTACCTggGCAGGCAATAGC-3′
